# A systems genomics and genetics approach to identify the genetic regulatory network for lignin content in *Brassica napus* seeds

**DOI:** 10.3389/fpls.2024.1393621

**Published:** 2024-06-05

**Authors:** Wentao Zhang, Erin E. Higgins, Stephen J. Robinson, Wayne E. Clarke, Kerry Boyle, Andrew G. Sharpe, Pierre R. Fobert, Isobel A. P. Parkin

**Affiliations:** ^1^ Aquatic and Crop Resource Development, National Research Council of Canada, Saskatoon, SK, Canada; ^2^ Saskatoon Research and Development Centre, Agriculture and Agri-Food Canada, Saskatoon, SK, Canada; ^3^ Global Institute for Food Security (GIFS), University of Saskatchewan, Saskatoon, SK, Canada; ^4^ Aquatic and Crop Resource Development, National Research Council of Canada, Ottawa, ON, Canada

**Keywords:** systems genomics, systems genetics, lignin, QTL, eQTL, regulatory network, seed quality, *Brassica*

## Abstract

Seed quality traits of oilseed rape, *Brassica napus* (*B. napus*), exhibit quantitative inheritance determined by its genetic makeup and the environment via the mediation of a complex genetic architecture of hundreds to thousands of genes. Thus, instead of single gene analysis, network-based systems genomics and genetics approaches that combine genotype, phenotype, and molecular phenotypes offer a promising alternative to uncover this complex genetic architecture. In the current study, systems genetics approaches were used to explore the genetic regulation of lignin traits in *B. napus* seeds. Four QTL (qLignin_A09_1, qLignin_A09_2, qLignin_A09_3, and qLignin_C08) distributed on two chromosomes were identified for lignin content. The qLignin_A09_2 and qLignin_C08 loci were homologous QTL from the A and C subgenomes, respectively. Genome-wide gene regulatory network analysis identified eighty-three subnetworks (or modules); and three modules with 910 genes in total, were associated with lignin content, which was confirmed by network QTL analysis. eQTL (expression quantitative trait loci) analysis revealed four cis-eQTL genes including lignin and flavonoid pathway genes, *cinnamoyl-CoA-reductase* (*CCR1*), and *TRANSPARENT TESTA* genes *TT4*, *TT6*, *TT8*, as causal genes. The findings validated the power of systems genetics to identify causal regulatory networks and genes underlying complex traits. Moreover, this information may enable the research community to explore new breeding strategies, such as network selection or gene engineering, to rewire networks to develop climate resilience crops with better seed quality.

## 1 Introduction

Understanding the genetic and molecular architecture underlying complex quantitative traits or phenotypic variation is the central goal of current biology (Kliebenstein, 2009). Changing transcript abundance arising from sequence polymorphism is one possible underlying molecular mechanism for complex traits (Rockman and Kruglyak, 2006). A wide range of complex traits within multiple species have been shown to be controlled by their underlying gene expression (Kliebenstein, 2009). A large-scale analysis of the relationship between complex diseases and known non-synonymous single nucleotide polymorphisms (SNPs) found that an increased number of SNPs did not find a significant number of associations, further indicating the potential role of variation in gene expression in controlling phenotypic traits (Burton et al., 2007). Together, these findings support the hypothesis that gene expression acts as the intermediate molecular phenotype, connecting genotypic variation to phenotypic variation (Rockman and Kruglyak, 2006).

The traditional quantitative trait loci (QTLs) mapping approach has been widely used to dissect the molecular mechanism underlying complex traits. However, most complex traits are controlled by multiple genes (Kliebenstein, 2009), and multiple QTLs with marginal effects (Mackay et al., 2009), which can be difficult to dissect with traditional QTL analysis. With the advent of advanced high-throughput technologies, including expression arrays and genome sequencing technologies, a new approach emerged to meet the challenge of complex traits, gene expression quantitative trait locus (eQTL) analyses. This approach is characterized by its combination of population genomic and quantitative genetics (Jansen and Nap, 2001). Briefly, QTL analysis is applied to the transcript abundances measured from a well-genotyped segregating population, allowing genetic loci (eQTL) controlling variation of each transcript to be identified on a genome-wide level (Keurentjes et al., 2007). A locus of sequence polymorphism affecting gene expression that is tightly linked to the physical position of the gene itself is defined as a cis-eQTL; all other eQTLs act in trans (trans-eQTLs) (Brem et al., 2002). The heritability of transcript variation and the genetic effect on this variation can also be inferred from eQTL analysis. Studies from Arabidopsis (West et al., 2007; Keurentjes et al., 2007), yeast (Brem and Kruglyak, 2005), maize (Schadt et al., 2003), and barley (Potokina et al., 2007), revealed an uneven distribution of trans-eQTLs with some genomic regions identified as eQTL hotspots (Schadt et al., 2003), enriched with numerous trans-eQTLs. Identification and dissection of eQTL hotspots may help to identify underlying master regulators or pleiotropic genes, and concomitantly the affected downstream genes or biological pathways. Thus, global eQTL analysis has the potential to yield great insight into the complex genetic architecture of transcript variation, and when linked to phenotypic variation (pQTLs), molecular mechanisms underlying complex traits can be directly inferred.

Several *in silico* large-scale data analyses have demonstrated the power of gene co-expression networks in dissecting a range of biological problems such as secondary wall biosynthesis (Persson et al., 2005), glucosinolate accumulation (Hirai et al., 2007), and seed germination and dormancy (Bassel et al., 2011) at the systems level. Gene regulatory networks were also hypothesized to constitute important information that can be inferred from eQTL data (Jansen and Nap, 2001); yet, this component has often been overlooked. Studies on seed development of Arabidopsis demonstrated that seed filling was controlled by a dynamic and distinct developmental transcriptional program, and genes involved in central carbon transport and multiple other metabolic pathways were implicated (Ruuska et al., 2002). However, most eQTL analyses have been limited to single gene-level analysis.

Fibre in seed meal negatively affects digestion and processibility for animal feed, and reducing the seed fibre content in oilseed rape could result in increased seed oil and protein content (Wittkop et al., 2009; Snowdon et al., 2010; Liu et al., 2012, 2013; Yu et al., 2016; Behnke et al., 2018; Miao et al., 2019; Chao et al., 2022; Yusuf and Möllers, 2024). Therefore, the low fibre trait was identified as a high-value target for canola (*Brassica napus*) breeding programs. Crude fibre is mainly comprised of lignin, including neutral detergent fibre (NDF), acid detergent fibre (ADF), acid detergent lignin (ADL), and polysaccharides including cellulose and hemicellulose (Van Soest et al., 1991; Wittkop et al., 2012). Genetics studies revealed a few major QTLs for lignin traits concentrated on chromosomes A09, C09, and C05 (Badani et al., 2006; Liu et al., 2012, 2013; Stein et al., 2013; Yu et al., 2016; Behnke et al., 2018; Miao et al., 2019; Chao et al., 2022). Lignin biosynthetic pathway(s) are complex. Forward and reverse genetics and biochemical analyses have identified more than 600 involved genes, with a subset of key enzymes that include *phenylalanine ammonia-lyase1* (*PAL1*) and *PAL2*; *cinnamate 4-hydroxylase* (*C4H*); *4-coumarate: CoA ligase1* (*4CL1*) and *4CL2*; *caffeoyl-CoA O-methyltransferase1* (*CCoAOMT1*); *cinnamoyl-CoA reductase1* (*CCR1*); *ferulate 5-hydroxylase* (*F5H1*); *caffeic acid O-methyltransferase* (*COMT*); and *cinnamyl alcohol dehydrogenase6* (*CAD6*) (Vanholme et al., 2010). Previous studies also reported that QTLs for seed colour colocalize with lignin content in *B. napus* seeds (Badani et al., 2006; Liu et al., 2012, 2013; Yu et al., 2016; Chao et al., 2022). In Arabidopsis, genes in the flavonoid pathway, including those from the *TRANSPARENT TESTA* (TT) family (eg*. TT1-TT19*) and transcription factor complex MYB-bHLH-WD40, are key regulators controlling seed colour (Lepiniec et al., 2006; Xu et al., 2014, 2015). The biosynthetic pathways of lignin and pigments share the same precursors (Vanholme et al., 2012), indicating that the two complex biosynthetic pathways may act together to control both lignin and seed colour, and possibly other quality traits in *B. napus* seeds.

As Arabidopsis is a very close relative of *B. napus* within the Brassicaceae family, it is reasonable to infer that seed quality traits of *B. napus*, e.g., lignin, are also controlled by interactions of multiple metabolic and biological pathways. Therefore, a systems genetic approach that combines quantitative genetics, regulatory network analysis, and eQTL analysis would be more appropriate to investigate seed quality traits within *B. napus (* Kliebenstein, 2009 and Marshall-Colón and Kliebenstein, 2019; Kliebenstein, 2020). In the present study, we aimed to fully exploit the power of this approach to decipher the regulatory network underlying lignin traits in *B. napus* seeds.

## 2 Materials and methods

### 2.1 Plant materials

The *B. napus* lines used in this study include the two parental lines DH12075, a Canadian spring-type doubled haploid canola line (generated by Dr. Gerhard Rakow and Dr. Ginette Séguin-Swartz, Agriculture and Agri-Food Canada), and PSA12, a resynthesised *B. napus* line (generated by Dr. Monica Beschorner and Dr. Derek Lydiate, Agriculture and Agri-Food Canada), and 96 doubled haploid segregating (SG) lines generated from the cross of these two parental lines. PSA12 was developed from an interspecific cross between *B. oleracea ssp. alboglabra* line A12DH and *B. rapa* line Parkland Sunshine, and DH12075 was derived from a cross between varieties Cresor and Westar (Mayerhofer et al., 2005).

The developing seeds at 21 days post-anthesis were collected from *B. napus* lines grown under field conditions at the AAFC Saskatoon Research Farm in 2009. This time point was selected because approximately 20 days after flowering (DAF) had previously been identified as a critical stage for cell proliferation and oil deposition (O’Hara et al., 2002; Dong et al., 2004). Total RNA was extracted from the developing seeds using a method modified from that of Oñate-Sánchez and Vicente-Carbajosa (2008) and described in detail by Parkin et al. (2010). Seeds from field grown plants from two trials (2009 and 2010) were harvested for seed quality analysis.

### 2.2 Seed quality analysis

Seed quality traits including oil content (OilHD), protein, lignin content of NDF, ADF, and ADL, iodine value (IV), hydrogenated density (HD) total glucosinolate, and chlorophyll were estimated by near-infrared reflectance (NIR Systems Model 6500, FOSS, Eden Prairie, MN) following methods described by Yu et al. (2016). Results are presented as percentage of whole seed dry matter with zero moisture. The system was calibrated for oil content using the method of Raney and Serblowski (2007), using samples hexane extracted following the method described by Raney et al. (1987). Fatty acid composition of the extracted oil samples was also analysed using the Agilent 6890 GC-FID (Agilent Technologies, Santa Clara, CA) (OilEx).

### 2.3 Array processing and data analysis

cRNAs were amplified from total RNA samples (2 µg) and labelled with either cy3 or cy5 using the Quick Amp labelling kit, Two Colour (Agilent, Catalogue: 5190-0444) according to the manufacturer’s instructions, followed by purification with the Qiagen RNeasy Mini Kit (Qiagen, Catalogue 74104) and quantification with a NanoDrop ND-1000. Purified cRNAs (2 µg) were fragmented and hybridized to the Agilent 4x44K *Brassica* arrays (Parkin et al., 2010) for 17 h at 65°C with a rotation of 10 rpm according to the manufacturer’s protocol. Arrays were washed with the Agilent Gene Expression Washing buffers according to the manufacturer’s protocol and scanned with GenePix 4000B as described by Parkin et al. (2010). Four and two biological replications were performed for the parental lines and the SG lines, respectively, with dye-swaps applied to the biological replicates to minimize dye effects from the two colour systems (Lee et al., 2004).

The raw array datasets were extracted with Gene Pix Pro 6.0, and were firstly normalized by the lowess (Yang et al., 2002) method within arrays, followed by the quantile (Bolstad et al., 2003) method between arrays, using the R package Limma (Smyth, 2005). The normalized datasets of the SG lines were averaged across replications. Probes with intensities over 20 and with a frequency less than 30 percent from our datasets were treated as non-significant signal probes and removed, resulting in a final total of 29,752 quality probes. Finally, the normalized gene levels lower than 20 were adjusted to 20 as background intensity. The log2 transformed normalized data were treated as gene expression traits (e-traits) and were used to perform eQTL analysis.

### 2.4 Differential gene expression analysis of parental lines DH12075 and PSA12

Normalized and adjusted data of parental lines with four biological replications were imported to GeneSpring GX 10.0 (Agilent Technologies) and analysed differential gene expression with Student’s t test. The p-value was adjusted with Benjamini-Hochberg’s method and controlled at FDR = 0.05. Genes with adjusted p-value < 0.05 and fold changes equal or larger than 2.0 were claimed as significantly differentially expressed genes.

### 2.5 Genotyping and linkage map construction

Genomic DNA was extracted with a modified CTAB method and genotyped with simple sequence repeat (SSR) markers developed at the Saskatoon Research Centre, Agriculture and Agri-Food Canada (http://aafc-aac.usask.ca/BrassicaMAST/). The SSRs were analysed using fluorescently labelled tail PCR amplifications in 384-well format on an Applied Biosystems 3730xl. Genotype data was imaged using genographer (available at http://www.genographer.com/), and polymorphic loci were scored visually. The SNP genotyping data of the SG population were generated by the Brassica 60 K Infinium array from Clarke et al. (2016).

### 2.6 Linkage map construction and QTL mapping

Genetic maps were developed with Mapdisto (Lorieux, 2012) using 694 SSR markers and 1266 SNP (binned map) markers with a cut-off recombination value of 0.3, and a threshold logarithm of odds (LOD) of 6.5. Physical positions of mapped markers were retrieved by BLASTN-searching SNP flanking sequences against the Darmor-bzh reference genome (Chalhoub et al., 2014).

QTL analysis was performed by composite interval mapping (CIM) as implemented in WinQTL Cartographer (Wang et al., 2007) with a walking speed of 1 cM and a window size of 10 cM. The significance of a QTL was claimed at the threshold of 5% significance level by the 3000-permutation test in WinQTL Cartographer (Churchill and Doerge, 1994; Basten et al., 2004; Wang et al., 2007). QTL within an interval of 10 cM were merged into one QTL, represented by the QTL peaks. QTL were visualized by the R package LinkageMapView (Ouellette et al., 2018).

### 2.7 Gene co-regulatory network construction

Normalized and filtered transcripts were used to construct a weighted gene co-regulatory network (WGCNA) with the step-by-step method implemented in the WGCNA R package (Zhang and Horvath, 2005; Langfelder and Horvath, 2008). The soft-thresholding power function analysis was applied correlation of adjacency of genes with the power value from 1 to 20 (**Supplementary Figure S1**). The optimized power value was 12, at which an approximal scale-free network was reached (**Supplementary Figure S1**). With this value, the adjacency matrix was transformed into a topological overlap matrix (TOM). Gene hierarchical clustering tree was generated by the dynamic tree-cutting method, hcluster. Modules with eigenvalue similarity >= 0.75 were merged, and modules with less than 30 genes were removed. Modules and their relationship to external traits were also obtained through the method within this package (Langfelder and Horvath, 2008). The *B. napus* seed network was generated with a hard cut-off value of |r|= 0.75 which made the network follow a roughly power-law distribution (Ravasz et al., 2002; Barabási and Oltvai, 2004). Identified gene interactions were imported into Cytoscape (Shannon et al., 2003) version 2.7.0 for visualization and additional analysis.

### 2.8 Module-trait association and module QTL (network QTL) analysis

The gene expression profile in each module was characterized by the eigenvalues of each module, and is referred to here as the module eigengene value (ME). The module-trait association was performed by Pearson correlation analysis between MEs and seed quality traits implemented within the WGCNA package. Eigengene value of the network can extract the key information (features) of the larger number of correlated genes within the network, and retain its most important variance of the network dataset, and reduce its dimensionality (Zhang and Horvath, 2005; Langfelder and Horvath, 2008). Thus, the ME that captures the summarized variance of the regulatory network can be treated as a classical phenotypic trait representing the correlated gene expression patterns of the whole network. Subsequently, QTL mapping of MEs (network QTL) was performed as described above (section 2.6).

### 2.9 Gene annotation, functional and enrichment analysis

ESTs of array probes were used in BLASTP (E-value <1e−5, coverage >70%, identity >70%) against the *Arabidopsis* Information Resource protein database (TAIR 10). The BLASTP output and the *Arabidopsis* functional annotations were parsed with in-house scripts to derive functional annotation and relevant GO terms.

Gene function terms were characterized with Mapman (Usadel et al., 2005) ontology terms and mapped into different biological pathways with tools embedded in the Mapman software (Usadel et al., 2005). Gene function enrichment analysis within detected modules was performed with the Fisher’s exact test and significance was claimed at p < 0.05.

### 2.10 eQTL analysis

The log transformed gene expression values were treated as a trait and analysed with the composite interval mapping (CIM) (Zeng, 1994) module “zmapqtl”, which was implemented in QTL Cartographer version 1.17 (Basten et al., 2004) under the Linux system, with a walking speed of 1 cM and a window size of 10 cM. Due to the limited capacity of the computation, a global permutation threshold (GPT) described by West et al., 2007, was performed to obtain a threshold criterion for declaring a statistically significant eQTLs. The GPT value (p = 0.05) equals a LOD score of 4.75 for this eQTL analysis. eQTLs within 10 cM intervals were merged into one QTL represented by the QTL peak.

The sequence of the array probes was also used with BLASTN (E-value < 1e-10) against the Darmor-bzh reference genome (Chalhoub et al., 2014) and physical positions of the best-hit were retrieved. The eQTL was defined as cis- if the gene and the marker linked to it co-localized within a physical interval of 4 Mb; otherwise, they were defined trans-eQTLs. According to this definition, cis- and trans-eQTL of *B. napus* were identified.

## 3 Results

### 3.1 Characterization of QTL for lignin content

**Figure 1:**
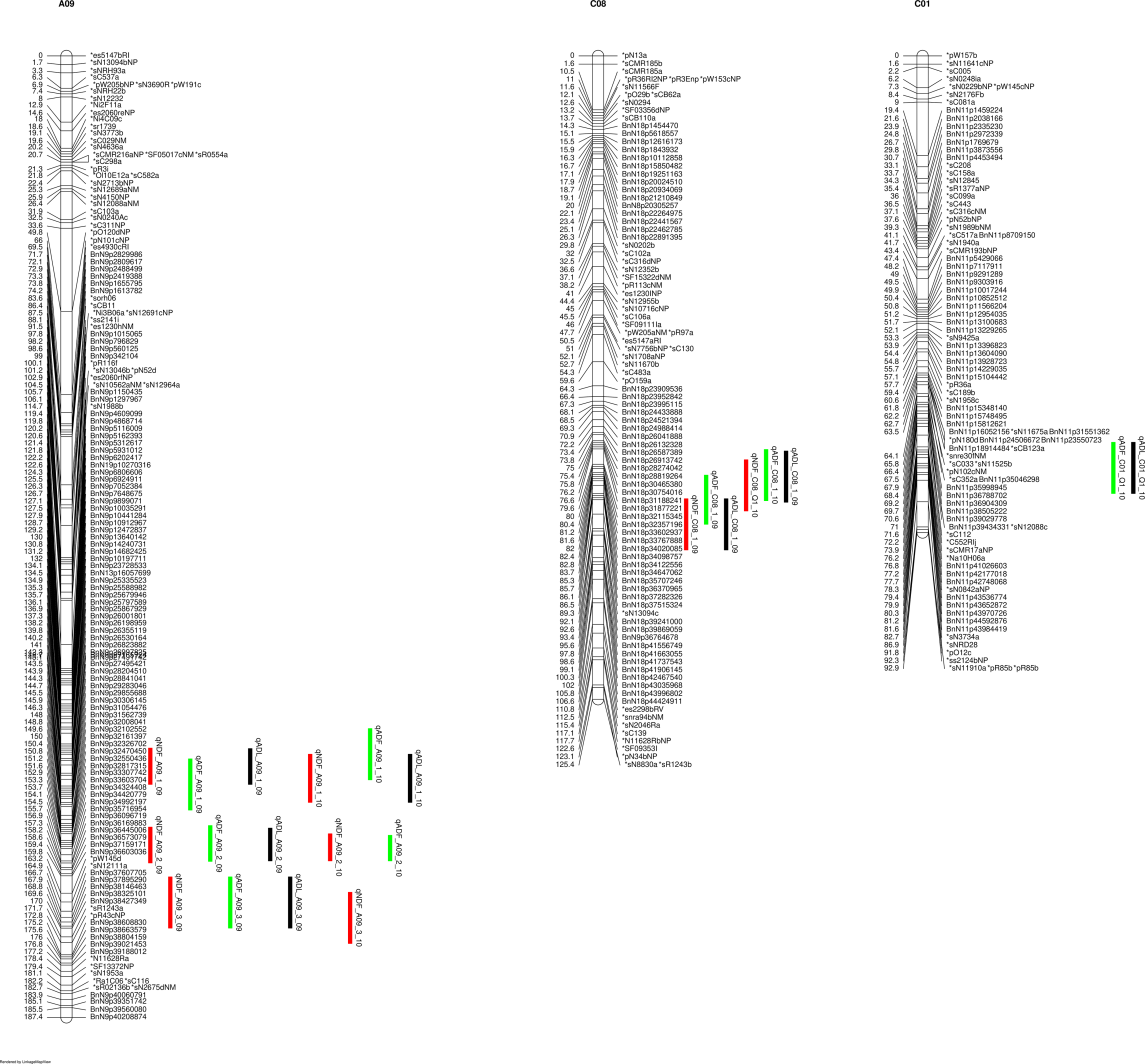
QTL for lignin content on identified on chromosomes A09 and C08 of the SG genetic map. NDF (neutral detergent fibre), indicated with red bars; ADF (acid detergent fibre), indicated by green bars, ADL (acid detergent lignin), indicated with black bars. 09 and 10 indicate the years 2009 and 2010.

The lignin contents on the seeds of this DH population exhibited a normal distribution, assessed using the Shapiro-Wilk test (p < 0.05) (**Supplementary Figure S2**). The parental line DH12075 consistently showed higher lignin contents than the resynthesized PSA12 in different environments (**Supplementary Figure S2**). We consistently identified major QTLs for lignin content NDF, ADF, and ADL from two-years of field trials, on chromosomes C08 and A09 (**Figure 1**), while a minor QTL from C01 was also identified in 2010. The NDF, ADF, and ADL shared the same QTLs on A09 and C08 (**Figure 1**), and since it has been suggested that investigation of ADF or NDF alone was sufficient to dissect the quantitative traits of fibre and lignin content (Cardinal et al., 2003), we therefore decided to use ADF as a representative trait to explore the genetic architecture underlying seed lignin traits. We defined three QTLs on A09: qLignin_A09_1 (comprised of qNDF_A09_1, qADF_A09_1 and qADL_A09_1); qLignin_A09_2 (from qNDF_A09_2, qADF_A09_2 and qADL_A09_2), and qLignin_A09_3 (from qNDF_A09_3, qADF_A09_3 and qADL_A09_3). The QTL on C08 was defined as qLignin_C08 (consisting of qNDF_C08, qADF_C08 and qADL_C08). qLignin_A09_1 spanned an interval of 130-145 cM and was located at 19-25 Mb on A09. It conferred effects with R^2^ of 7% in 2009 and 12% in 2010. The qLignin_A09_2 conferred the largest effects in the two-year test, and localized between 145-160 cM with a physical interval of 25-30 Mb. The qLignin_A09_3 localized between 160-170 cM with an interval of 30-33 Mb on A09. The qLignin_C08 was found in the interval of 82-92 cM with the physical interval of 30-40 Mb on C08. The QTL qLignin_A09_1, qLignin_A09_3, and qLignin_C08 conferred a similar magnitude of effect on lignin content and with ~3-5% less than the qLignin_A09_2. Synteny analysis identified qLignin_A09_2 and qLignin_C08 are homologous QTL from the A and C subgenomes, respectively (**Supplementary Figure S3**). The alleles for all QTL on A09 that confer the higher level of lignin content are from parental line DH12075, while the alleles for the higher lignin QTL on C08 are from the synthetic parental line, PSA12.

### 3.2 Identification of genetic regulatory networks underlying lignin content

**Figure 2:**
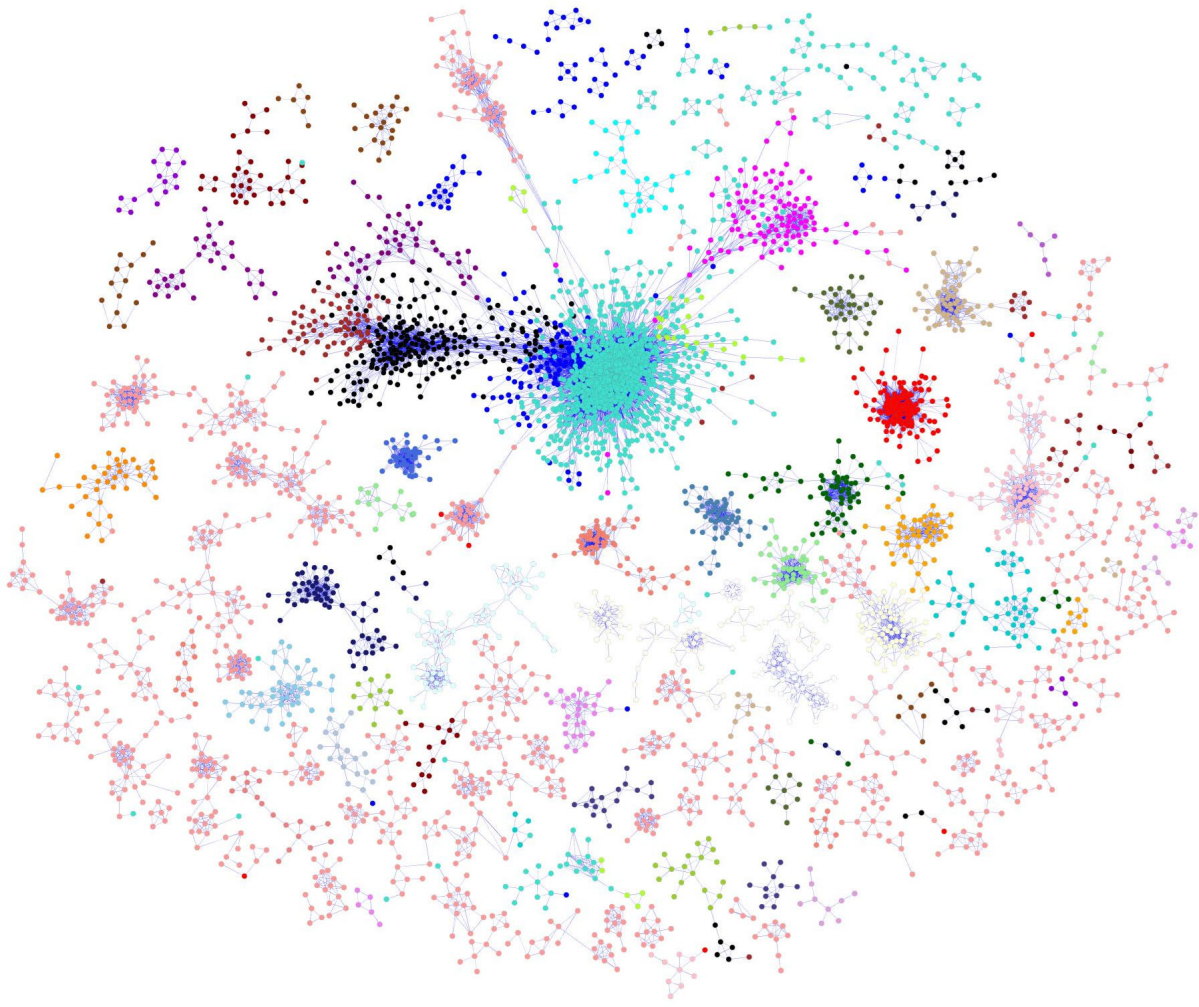
*B. napus* co-regulatory network. Nodes are probe sets from the customized *B. napus* array. The node colours depict the different modules identified from the network. Edges indicate significant co-expression between probe sets above a hard threshold. In order to improve the clarity of the display, only correlation coefficient (|r|>= 0.85) were shown on the network.

A total of 29,725 transcripts were used to build a co-regulatory network with WGCNA. The final co-expression network consisted of 12,670 nodes connected by 468,974 edges, containing ~60% of the information from the input datasets. The entire network was partitioned into 83 distinct gene modules (or subnetworks). A global view of the co-regulatory network with modules coloured distinctly on the network is shown in **Figure 2**. The number of genes within these subnetworks ranged from 30 to 7,303 genes.

**Figure 3:**
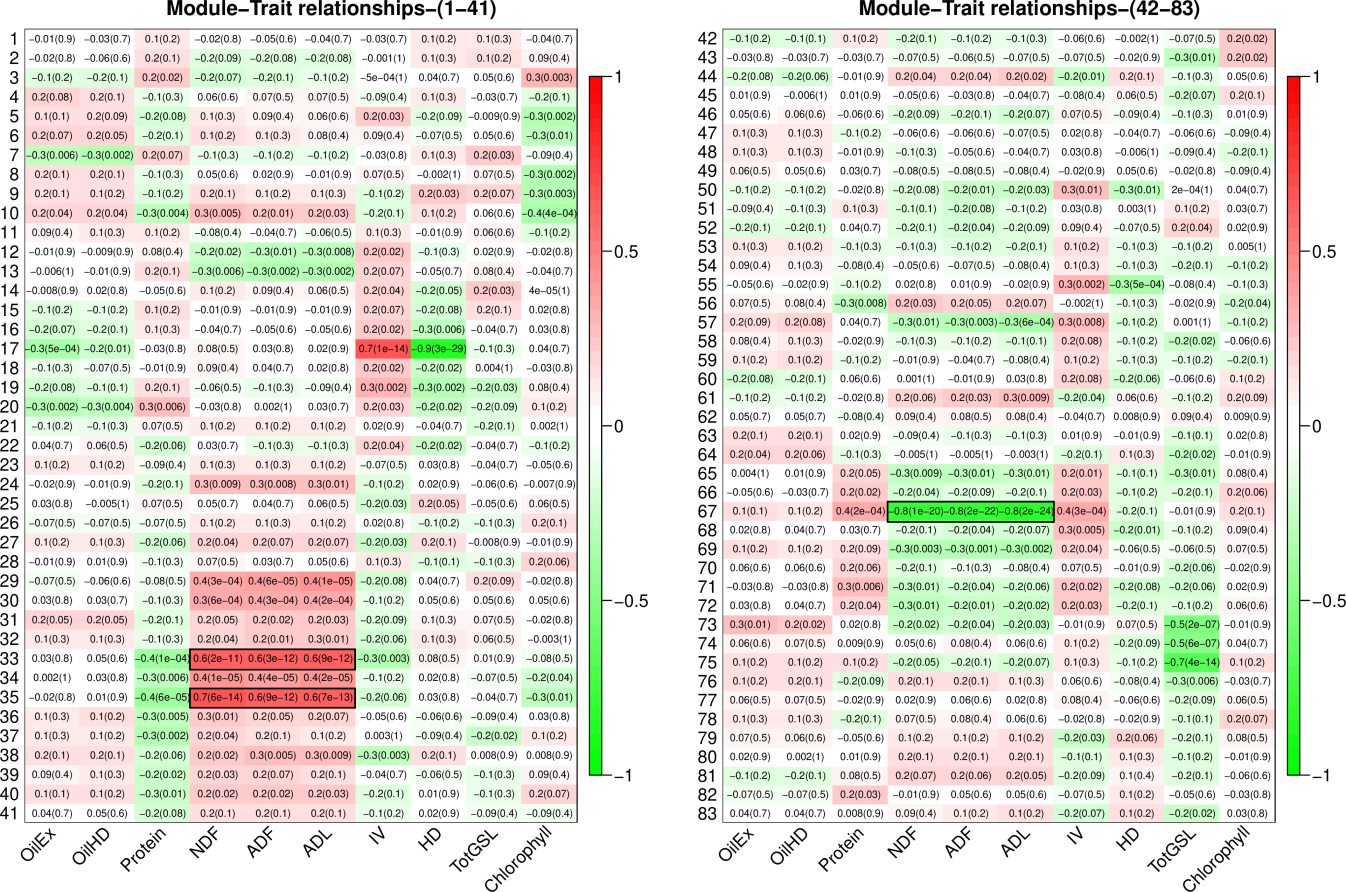
Module-trait relationship between 83 modules and seed quality traits. Traits and rows represent the modules. Correlation co-efficient and p-value (in parenthesis) were shown in each cell. Correlation between modules and seed quality traits was presented by colours ranging from red (high positive correlation) to green (high negative correlation). Evaluated seed quality traits include oil content (OilHD, measured by NIR; OilEx, determined by GC-FID), protein, lignin content of NDF (neutral detergent fibre), ADF (acid detergent fibre), ADL (acid detergent lignin), iodine value (IV), hydrogenated density (HD), total glucosinolate (TotGSL), and chlorophyll.

Module-trait association analysis was performed to identify regulatory networks associated with seed quality traits in *B. napus* seeds; including oil content, protein, NDF, ADF, ADL, IV, glucosinolate, hydrogenated density, glucosinolate, and chlorophyll. Module 33 and module 35 were identified as significantly positively associated with NDF, ADF, and ADL, while module 67 was significantly negatively correlated with these three forms of lignin (**Figure 3**). As shown in **Figure 3**, the correlation coefficients between ADF and module 33, module 35, and module 67 were 0.6 (p-value: 3E-12), 0.6 (p-value: 9E-12), and -0.8 (p-value: 2e-22), respectively.

**Figure 4:**
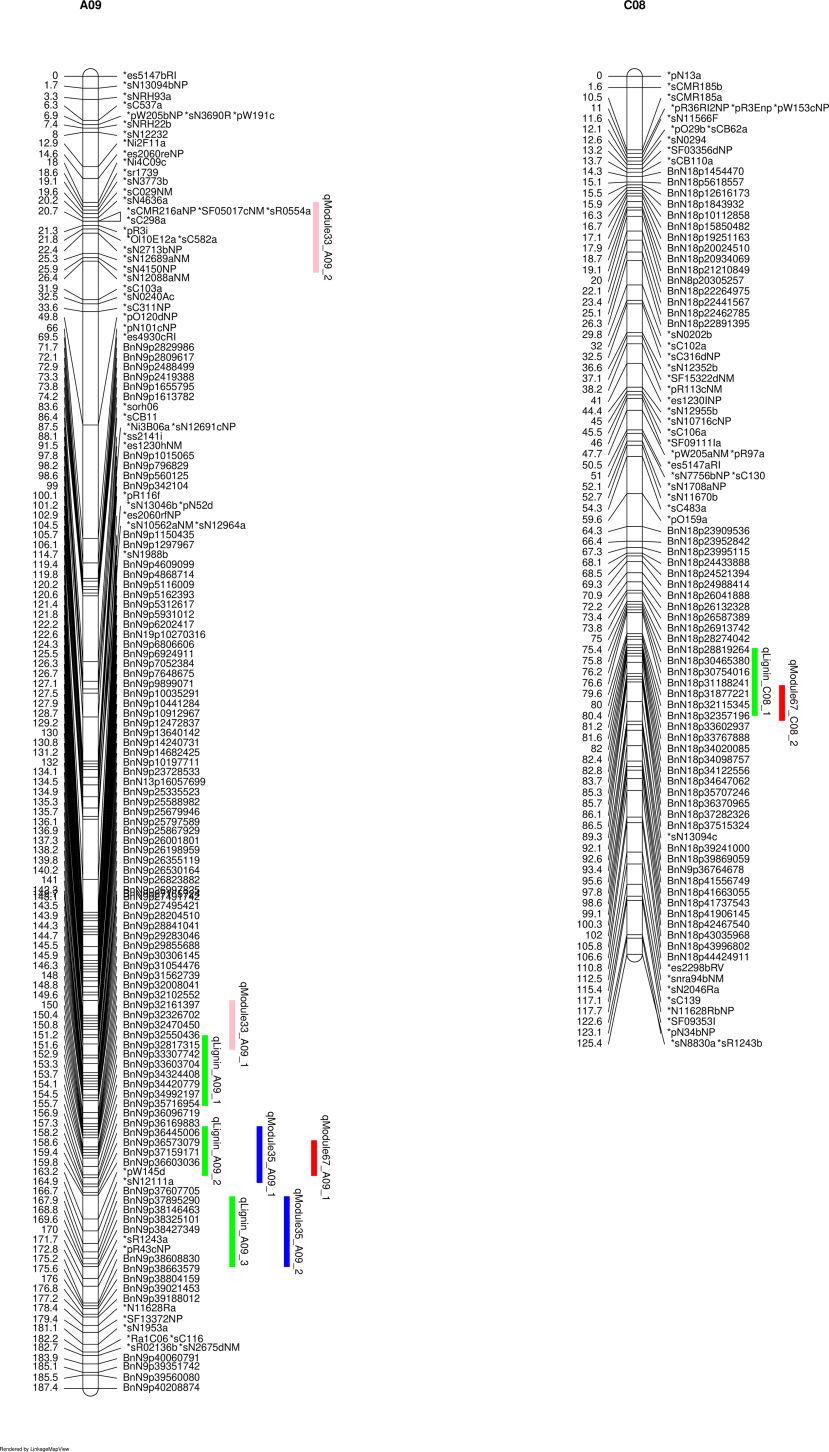
Linked lignin phenotype QTLs (pQTLs) to module QTLs (mQTLs). qLignin are lignin QTL, and qModule are module QTL.

**Figure 5:**
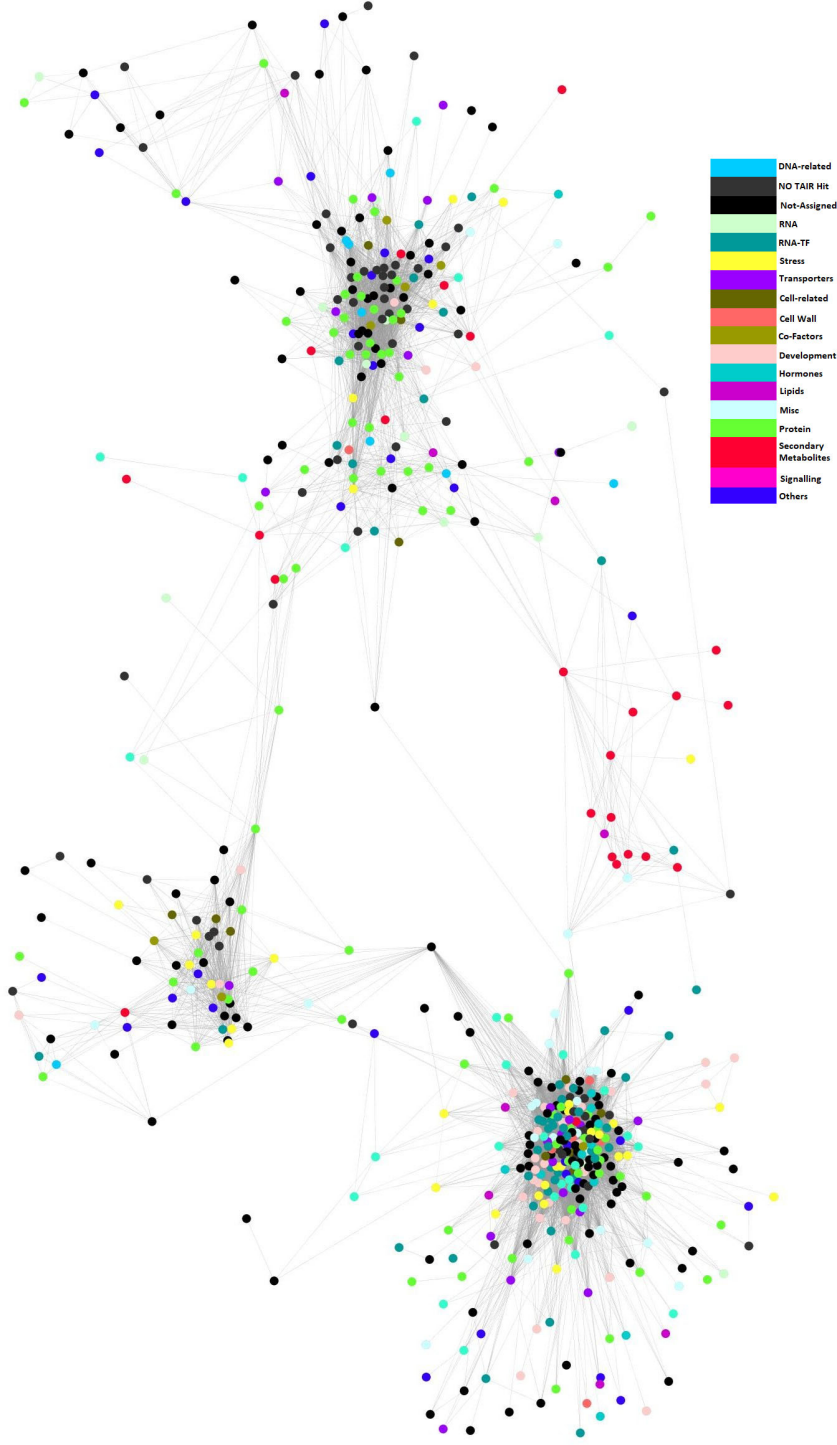
Co-regulatory network associated with lignin. Nodes are transcripts identified from modules 33, 35 and 67. Edges indicate significant co-expression between probe sets above a hard threshold. Node colours depict different Mapman ontology terms (legend right).

To validate that these modules are causal networks of lignin traits, a network QTL mapping approach was applied, utilizing linkage mapping to identify the genetic locus that controlled these modules. As shown in **Figure 4**, the QTL for module 33, qModule33_A09_1 overlapped with lignin pQTL qLignin_A09_1 on A09. The two QTLs for module 35, qModule35_A09_1 and qModule35_A09_2, colocalized with qLignin_A09_2 and qLignin_A09_3, respectively (**Figure 4**). The qModule67_A09_1 colocalized with qLignin_A09_2 and qModule_67_C08_1 collocated with qLignin_C08 (**Figure 4**). To further explore these regulatory networks, genes were extracted from these three modules and a sub-network built, which we termed the lignin causative network (**Figure 5**).

### 3.3 Gene function and enrichment analysis of the genetic regulatory network for lignin content

From the metabolic overview map, the major metabolite pathways that corresponded with the change of lignin content were characterized by genes involved in secondary metabolism, lipid biosynthesis, and cell wall modification (**Supplementary Figure S4**). Gene function enrichment analysis revealed that the lignin causal network was enriched with genes related to secondary metabolites (p< 1.8-E04) (**Supplementary Table S1**). These secondary metabolism-related genes were mapped mainly on the flavonoid, phenylpropanoids, lignin, shikimate, and mevalonate (MVA) pathways (**Supplementary Figure S5**). Major genes involved in lignin and flavonoid pathways, including *BANYULS (BAN*), *CCR1*, *C4H*, *PAL2*, and the *TT*-related genes*, TT3*, *TT4*, *TT6*, *TT7*, *TT8*, *TT18*, *TT19*, were found positively correlated with lignin as shown in **Supplementary Table S2**, **Supplementary Figure S6**.

Stress-related genes were also enriched in the regulatory network (p-value: 0.01) (**Supplementary Table S1**); therefore, we explored the patterns of transcripts involved in abiotic and biotic stress. An interesting change in gene expression patterns associated with biotic stress in response to the change of seed lignin was observed. Almost all secondary metabolite-related genes involved in biotic stress were positively correlated with the change of lignin, while many of the major genes involved in biotic stress, including WRKY transcription factors (*WRKY25* and *WRKY33*), ethylene-related AP2 transcription factors, ethylene-related genes, and MAP kinases, were negatively correlated with lignin accumulation (**Supplementary Table S2**, **Supplementary Figure S7**).

If the genes are causal for the trait, it can be hypothesized the eQTL of the gene will colocalize with the trait QTL (pQTL). Prioritized causal genes will be shown as cis-eQTLs, in which the gene controls the phenotypic variation by mediating its expression via cis-regulatory sequence variation. eQTL analysis of the 910 genes within the lignin regulatory network found 163 cis-eQTL and 491 trans-eQTL underlying the three lignin QTL of the A09 chromosome (**Supplementary Table S3**). There were 14 cis- and 136 trans- eQTL identified for the C08 lignin QTL (**Supplementary Table S4**). The lignin gene *CCR1* and seed colour genes *TT4*, *TT6*, and *TT8* were identified as cis-eQTLs that colocalized with the lignin QTL on A09. The key lignin genes, *PAL2* and *C4H*, and seed colour genes *TT3*, *TT7*, *TT18*, *TT19*, and *BAN* were found to be trans-eQTLs (**Supplementary Tables S3**, **S4**), and none of the cis-eQTLs of lignin and flavonoid genes from C08 QTL were found above the significance threshold value. Gene expression analysis between the two parental lines found that of the genes known to be important to the lignin pathway, only *CCR1* was significantly differentially expressed between the parental lines (**Supplementary Table S5**). DH lines with the eQTL of *CCR1* (based on the DH12075 marker type at the eQTL peak BnN9p34992197) have significantly higher group means of gene expression than lines without the eQTL (**Supplementary Figure S8**). The seed colour genes *TT8*, *TT6* and *TT19* were statistically differential expressed, but with a fold change of gene expression level between 1.5 to 2.0 (**Supplementary Table S5**). Lignin content was significantly associated with the expression of these genes with cis-QTL, *CCR1*, *TT6*, and *TT8* (**Supplementary Figure S9**).

## 4 Discussion

Complex traits or quantitative traits are typically controlled by complex genetic architecture consisting of hundreds to thousands of genes (Kliebenstein, 2009). These genes form a genetic regulatory network that controls the trait, which challenges the single gene or traditional QTL analysis. Therefore, we utilized a systems genetic approach that integrates the quantitative genetic, gene co-regulatory network, network-QTL, and eQTL analysis to uncover the causal regulatory network underlying lignin content in *B. napus* seeds.

Three major QTLs were identified on A09 spanning a physical interval of approximately 13 Mb. All three QTLs conferred stable and relatively large effects on lignin content. QTLs for NDF, ADF, and ADL colocalized at the same genetic locus, indicating these three components of lignin shared the same genetic architecture. Numerous QTLs for seed quality traits including seed colour, oil, protein, and fibre were previously identified on the A09 chromosome (Wittkop et al., 2009; Liu et al., 2012, 2013; Yu et al., 2016; Behnke et al., 2018; Miao et al., 2019; Chao et al., 2022; Yusuf and Möllers, 2024), indicating that A09 is a hotspot that harbours several genes for these seed quality traits. However, this study and all previous research have used a relatively small population size, from ~100 to 300 lines, making it challenging to precisely identify QTLs within this hotspot. Future, larger and advanced-structured genetic populations could be used to dissect this hotspot. In addition, a stable and large effect homologous QTL to the qLignin_A09_2 was identified on C08, indicating both the A and C genome contributed to the variation of lignin content in our population. Currently, limited studies, including Badani et al. (2006) and Chao et al. (2022), have reported the QTL on C08 for seed colour and lignin content. The relative simplicity of the C08 QTL, compared to A09, makes it a better target to uncover genes underlying lignin QTL in the future. Furthermore, since the C08 QTL is homologous to the A09 lignin QTL, uncovering its genetic architecture may also shed light on the A09 hotspot.

The genome-wide transcriptome datasets for developed seeds of the SG population, allowed a seed development biological network to be successfully built (**Figure 2**). Biological network construction assumes that it will follow the topological scale-free property, in which the distribution of the nodes and edges follows a power-law distribution (Barabási and Oltvai, 2004). Although definitions of the properties of biological networks is still under discussion, the seed developmental regulatory network did exhibit the expected scale-free topology, described as a small number of nodes with a large number of connections, while most nodes have few connections (**Figure 2**). The distribution of the nodes and edges in our network exhibited a power-law distribution, supporting its topological free property, and was in agreement with the network property discovered in Arabidopsis (Atias et al., 2009; Mao et al., 2009), rice (Ficklin et al., 2010; Ficklin and Feltus, 2011) and maize (Ficklin and Feltus, 2011).

The structure of genome-wide co-expression networks are hierarchical with modular units (Aoki et al., 2007). Given that genes with similar biological functions are typically co-expressed together and formed functional modules, disassembling biological networks into functional modules or clusters is more informative and practical for biologists to interpret the network (Mutwil et al., 2010). Here, the seed network was divided into 83 computational biological modules. If phenotype variation is attributed to the variation of genes within the module, a correlation should be observed between modules and phenotypes. This was the case for two modules (modules 33 and 35) that were significantly positively correlated with lignin, and one (module 67) that was negatively coordinated with lignin. With network QTL analysis, it was found that lignin QTLs (pQTLs) and module QTLs shared the same genetic loci on A09, supporting the suggestion that lignin variation was determined by the interactions of these three functional modules. These findings also support the hypothesis that entire plant networks can be inherited as a phenotypic trait (Marshall-Colón et al., 2019). Large-scale evaluation of 1,671 genes on agronomic performance in maize using a single gene engineering approach found that only one gene encoding a transcription factor, *zmm28*, showed promise, indicating the marginal effect of a single gene for the improvement of complex traits (Simmons et al., 2021). The defined lignin network could enable breeders to target a specific regulatory network and potentially use an engineering approach to rewire and fine-tune the network to improve crop performance.

Since modules 33, 35, and 67 were altogether associated with seed lignin, these three modules were assembled into one large lignin-related functional module, called “lignin causative network”, to perform further analysis. This network was enriched with secondary metabolism-related genes. One group of these genes includes *PAL2*, *C4H*, and *CCR1*, encoding three key enzymes controlling critical steps of the lignin pathway (Vogt, 2010). The other group, which mainly consists of flavonoid pathway genes, is involved in the accumulation of flavonoids in the seed coat, such as *TT3*, *TT7*, *TT6*, *TT8*, *TT18*, *TT19*, and *BAN*. All these genes are significantly and positively associated with seed lignin variation. Enrichment of these secondary metabolite genes indicated the high reliability of this causative regulatory network underlying lignin content.

eQTL analysis was performed on genes within this network to identify the causal genes underlying the network for lignin content. The lignin gene, *CCR1*, was detected as a cis-eQTL, while both *PAL* and *C4H* were found as trans-eQTLs. Flavonoid genes including *TT4*, *TT6*, and *TT8* were identified as cis-eQTLs, while the remaining *transparent testa* genes were trans-eQTLs. In addition, all these cis-eQTLs colocalize with the fibre QTL from chromosome A09, indicating they are prioritized as causal genes underlying this network (Kliebenstein, 2009). Liu et al. (2012) pinpointed *CCR1* from chromosome A09 as the candidate gene for ADF in *B. napus* seeds. Zhang et al. (2022) identified a CCR-like gene, *CCRL*, from C07 as one candidate gene for seed content in *B. napus*. *CCR1* has been found to encode the key enzyme for lignin accumulation in stems (Jones et al., 2001). The cis-controlled eQTL impacting the variation of *CCR1*, the co-localization with phenotype QTL, and the unique known gene from the monolignol pathway located on our lignin causative network as well as previous findings, indicated that *CCR1* is one of the causative genes underlying the seed lignin trait. Of the three cis-regulated flavonoid genes, *TT8* was found to be a key regulator for seed oil and colour in Arabidopsis and *B. napus* (Chen et al., 2014; Xu et al., 2014, 2015; Zhai et al., 2020; Zhang et al., 2022) and lignin in *B. napus* (Zhang et al., 2022). In addition, Zhai et al. (2020) found *TT8*, as a BHLH transcription factor, regulated both the flavonoid and lignin pathways. All these findings pinpoint *TT8* as another key regulator in this network. Li et al. (2023) reported that *TT4*, which encodes an enzyme acting at an earlier step of the flavonoid pathway and located on chromosomes A03 and C02, is key regulator for oil and protein content in *B. napus* seeds, while we identified *TT4* from A09 as playing a key role for lignin content. Therefore, it is hypothesized that multi-homologous and homoeologous *TT4* genes in different genotypes control seed quality traits. In addition, another earlier flavonoid biosynthetic gene *TT6* was identified as a potential new key regulator for lignin content in *B. napus*. Thus, the lignin traits associated with the A09 loci are controlled by a complex regulatory network with multiple genes.

Notably, we did not find any cis-eQTL from the key lignin and flavonoid genes associated with the homologous C08 lignin QTL. Network QTL analysis only found that the QTL of module 67 colocalized with the qLignin_C08 QTL. Genes for the development of *B. napus* seed are spatially and temporally regulated (Zhai et al., 2020). It is possible that the A and C genome coordinately regulate seed lignin content in a temporal manner, while the regulatory network built here, was only from one key time point, 21 days after flowering. We observed, under greenhouse conditions, that the A donor line of PSA12 flowered and matured earliest, followed by DH12075 and PSA12, while the C genome donor line of PSA12 flowered and matured very late (unpublished data), supporting this hypothesis, since positive alleles at the C08 locus were contributed by PSA12. A further spatial-temporal regulatory network built on seed development will be more powerful for dissecting the genetic architecture controlling seed quality traits and should be considered for future studies.

Another interesting finding of the lignin network was that it was enriched with biotic stress genes, including key transcript factors, *WRKY25*, *33* and *SIGMA FACTOR BINDING PROTEIN 1* (*SIB1*), which are involved in triggering resistance to the necrotrophic pathogen *Botrytis cinerea* in Arabidopsis (Andreasson et al., 2005; Zheng et al., 2006; Eulgem and Somssich, 2007; Pandey and Somssich, 2009; Lai et al., 2011). However, all these genes were significantly negatively associated with lignin accumulation, which is contradictory to the positive role of lignin on enhancing the resistance response (Vanholme et al., 2010). This paradoxical role of lignin in the plant defence mechanism compelled us to hypothesize that cell wall integrity plays a key role in this process. Reduced lignin affects the integrity of the wall which mimics disease infection, leading to the release of defence-related elicitors. When a normal plant is infected by disease, cell wall integrity is damaged and in response to this, the cell wall induces lignin biosynthesis to maintain its integrity. This theory is further supported by studies which have shown that cell wall damage directly triggers lignin biosynthesis (Denness et al., 2011).

It is worth noting a few limitations of the current study. First, the population size was small, and may not provide enough statistical power for identifying minor QTL. Second due to the limitation of array technology, many unknown genes within our causative network may be missing from our research. Third, this regulatory network was built only from one stage of seed development, and could not capture a complete temporal picture of development. New advanced structured genetic populations, and genome sequencing tools including long read and pangenome sequence analysis will empower these systems genetic approaches to uncover the genetic mechanism of complex traits.

## 5 Conclusion

An example of the power of systems genetics that combines quantitative genetic, regulatory network analysis, network QTL, and eQTL analysis to identify causal regulatory networks and causal genes for lignin traits was provided. Further insights were revealed into the complex genetic architecture that regulates lignin content, specifically that controlled by the locus on A09 of *B. napus*. The analyses provide more prioritized genes to target to improve seed quality trait in *B. napus*. Moreover, the research here may also provide a new avenue for crop communities to use regulatory networks to select or design crops with better performance and quality in a changing climate.

## Data availability statement

The data presented in the study are deposited in the NCBI data repository, accession number GSE268725.

## Author contributions

WZ: Data curation, Formal analysis, Investigation, Methodology, Validation, Visualization, Writing – original draft, Writing – review & editing. EH: Data curation, Formal analysis, Investigation, Methodology, Validation, Visualization, Writing – review & editing. SR: Data curation, Methodology, Writing – review & editing. WC: Data curation, Methodology, Writing – review & editing. KB: Formal analysis, Visualization, Writing – review & editing. AS: Conceptualization, Funding acquisition, Writing – review & editing. PF: Conceptualization, Funding acquisition, Investigation, Project administration, Supervision, Writing – review & editing. IP: Conceptualization, Funding acquisition, Methodology, Project administration, Resources, Supervision, Validation, Writing – original draft, Writing – review & editing.

## Supplementary material

Supplementary Table 1 Gene Ontology (GO) Enrichment analysis of the fibre related regulatory network.

Supplementary Table 2 Gene function, module, and its correlation with acid detergent fibre (ADF). Significant correlations between gene expression levels and ADF were claimed at p-value < 0.05.

Supplementary Table 3 eQTLs overlapping with pQTLs identified from the fibre related network on A09. cis and trans, eQTL defined by B. napus cv. Darmor-bzh reference genome. cis*, cis-eQTL defined by the DH12075 reference genome.

Supplementary Table 4 eQTLs overlapping with pQTLs identified from the fibre related network on C08. cis and trans, eQTL defined by B. napus cv. Darmor-bzh reference genome. cis*, cis-eQTL defined by the DH12075 reference genome.

Supplementary Table 5 Differential expressed genes within the fibre regulatory network between parental lines DH12075 (DH) and PSA12 (PSA). Up indicated higher gene expression level in DH12075. Corrected p-value: p-value was adjusted with Benjamini-Hochberg’s method and controlled at FDR (False Discovery Rate) = 0.05. Genes with more than 2- fold changes and corrected p-value < 0.05 were claimed as significantly differential expressed. Genes highlighted in red have a corrected p-value < 0.05, but a fold change between 2 - 1.5.

Supplementary Figure 1 Soft-thresholding powers test to identify the optimized value to fit the approximate scale free topology of the network. The left panel displayed the scale-free topology index (y-axis) with the tested soft-thresholding power (x-axis). The right panel displays the mean connectivity (degree, y-axis) with tested soft-thresholding power (x-axis).

Supplementary Figure 2 Distribution of lignin content in the years 2009 **(A-C)** and 2010 **(D-F)**. **(A, D)** acid detergent lignin (ADL); **(B, E)** neutral detergent fiber (NDF); **(C, F)** acid detergent fibre (ADF). Lignin contents of the parental lines PSA12 and DH12075 are highlighted with blue and red dashed lines, respectively. The normality of the distribution was assessed using the Shapiro-Wilk test, which yielded p values of **(A)** p = 0.001, **(B)** p = 0.032, **(C)** p = 0.003, **(D)** p = 0.001, **(E)** p = 0.0099, **(F)** p = 0.001, which all exceed the significance of p < 0.05.

Supplementary Figure 3 Synteny analysis of the lignin QTL qLignin_A09_2 and qLignin_C08. Synteny was analyzed and visualized with the Brassica napus Genome Browser (https://www.genoscope.cns.fr/brassicanapus/) with the Darmor-bzh reference genome (Chalhoub et al., 2014).

Supplementary Figure 4 Mapped transcripts from fibre network to the major metabolites map. Correlation between transcripts and seed ADF (acid detergent fibre) trait was presented by colors ranging from red (high positive correlation) to blue (high negative correlation).

Supplementary Figure 5 Dissection of transcripts on the major secondary metabolite pathways. Correlation between transcripts and seed ADF (acid detergent fibre) trait was presented by colors ranging from red (high positive correlation) to blue (high negative correlation).

Supplementary Figure 6 Mapped known genes to the lignin and flavonoid pathway. Correlation between transcripts and seed ADF (acid detergent fibre) were presented by colors ranging from red (high positive correlation) to blue (high negative correlation).

Supplementary Figure 7 Identification biotic stress signal pathway coordinated with the changes of lignin. Correlation between transcripts and seed (acid detergent fibre) trait was presented by colors ranging from red (high positive correlation) to blue (high negative correlation).

Supplementary Figure 8 Analysis of gene expression of double haploid (DH) lines with cis-eQTL of CCR1. The DH lines were separated into two groups based on the genotype of the eQTL peak marker *BnN9p34992197*: lines with the eQTL (DH12075 marker type) labeled in blue, and lines without the eQTL (PSA12 marker type) labeled with yellow. Student’s t-test was used to test the significance of means between groups at the significance level of p < 0.05.

Supplementary Figure 9 Pearson correlation analysis of trait-gene expression. The log2 transformed gene expression levels of **(A)**. *cinnamoyl-CoA-reductase (CCR1)*; **(B)**. *TRANSPARENT TESTA 6 (TT6)*; and **(C)**. *TRANSPARENT TESTA 8 (TT8)* are shown in the Y-axis, with acid detergent fibre (ADF) in the X-axis. Parental line DH12075 is indicated in green, PSA12 is indicated in red, and Double haploid (DH) lines are indicated by black dots. R, Pearson Correlation Coefficient; and p, p-value, significance level was claimed at p < 0.05.
