# Supplementary material for: A systems genomics and genetics approach to identify the genetic regulatory network for lignin content in Brassica napus seeds
Source: Front Plant Sci. 2024 Jun 5;15:1393621. doi: 10.3389/fpls.2024.1393621 (PMC11188405; doi:10.3389/fpls.2024.1393621)
Supplement: Supplementary Table 1 — Gene Ontology (GO) Enrichment analysis of the fibre related regulatory network. [file Table_1.docx]

| **Table S1: Gene Ontology (GO) Enrichment analysis of Fibre related regulatory network** | | | | |
| --- | --- | --- | --- | --- |
| **Bins** | **GO term** | **Observed Ratio** | **Expected Ratio** | **p-Value** |
| 1 | photosynthesis | 2/661 | 106/10930 | 0.72 |
| 2 | major CHO metabolism | 2/661 | 63/10930 | 1.00 |
| 3 | minor CHO metabolism | 5/661 | 73/10930 | 0.85 |
| 4 | glycolysis | 3/661 | 39/10930 | 0.71 |
| 5 | fermentation | 2/661 | 8/10930 | 0.22 |
| 7 | Oxidative pentose phosphate | 2/661 | 22/10930 | 0.60 |
| 8 | TCA / org- transformation | 1/661 | 47/10930 | 1.00 |
| 9 | mitochondrial electron- transport / ATP-synthesis | 4/661 | 88/10930 | 1.00 |
| 10 | cell wall | 12/661 | 168/10930 | 0.84 |
| 11 | lipid metabolism | 16/661 | 197/10930 | 0.43 |
| 13 | amino acid metabolism | 8/661 | 158/10930 | 1.00 |
| 15 | metal handling | 2/661 | 36/10930 | 1.00 |
| **16** | **secondary metabolism** | **21/661** | **144/10930** | **1.8-E04**** |
| 17 | hormone metabolism | 16/661 | 194/10930 | 0.43 |
| 18 | Co-factor and vitamin- metabolism | 3/661 | 29/10930 | 0.45 |
| 19 | tetrapyrrole synthesis | 1/661 | 27/10930 | 1.00 |
| **20** | **stress** | **31/661** | **300/10930** | **0.01**** |
| 21 | redox regulation | 7/661 | 117/10930 | 1.00 |
| 23 | nucleotide metabolism | 5/661 | 84/10930 | 1.00 |
| 26 | misc | 28/661 | 511/10930 | 1.00 |
| 27 | RNA | 67/661 | 1168/10930 | 1.00 |
| 28 | DNA | 10/661 | 271/10930 | 0.60 |
| 29 | protein | 102/661 | 1658/10930 | 0.85 |
| 30 | signalling | 39/661 | 459/10930 | 0.11 |
| 31 | cell | 20/661 | 373/10930 | 1.00 |
| 32 | microRNA, natural- antisense etc | 1/661 | 17/10930 | 1.00 |
| 33 | development | 22/661 | 254/10930 | 0.22 |
| 34 | transport | 28/661 | 433/10930 | 0.85 |
| 35 | not assigned | 202/661 | 3815/10930 | 0.23 |
| Bins: tree-structured bins of Mapman ontology; GO term: gene ontology term; Observed ratio: the percentage of the given GO term out of the total number of genes identified on the Fibre-related regulatory network (661 TAIR gene hits); Expected Ratio: the percentage of the given GO term out of the total number of genes identified from the whole regulatory network (10930 TAIR hit genes); Significance of GO term enrichment was tested by Fisher’s exact test at a significant threshold value of p-Value <0.05; “**” indicates significant GO term enrichment. | | | | |
