# Supplementary material for: A systems genomics and genetics approach to identify the genetic regulatory network for lignin content in Brassica napus seeds
Source: Front Plant Sci. 2024 Jun 5;15:1393621. doi: 10.3389/fpls.2024.1393621 (PMC11188405; doi:10.3389/fpls.2024.1393621)
Supplement: Supplementary Figure 1 — Soft-thresholding powers test to identify the optimized value to fit the approximate scale free topology of the network. The left panel displayed the scale-free topology index (y-axis) with the tested soft-thresholding power (x-axis). The right panel displays the mean connectivity (degree, y-axis) with tested soft-thresholding power (x-axis). [file DataSheet_1.pdf]

Figure S1

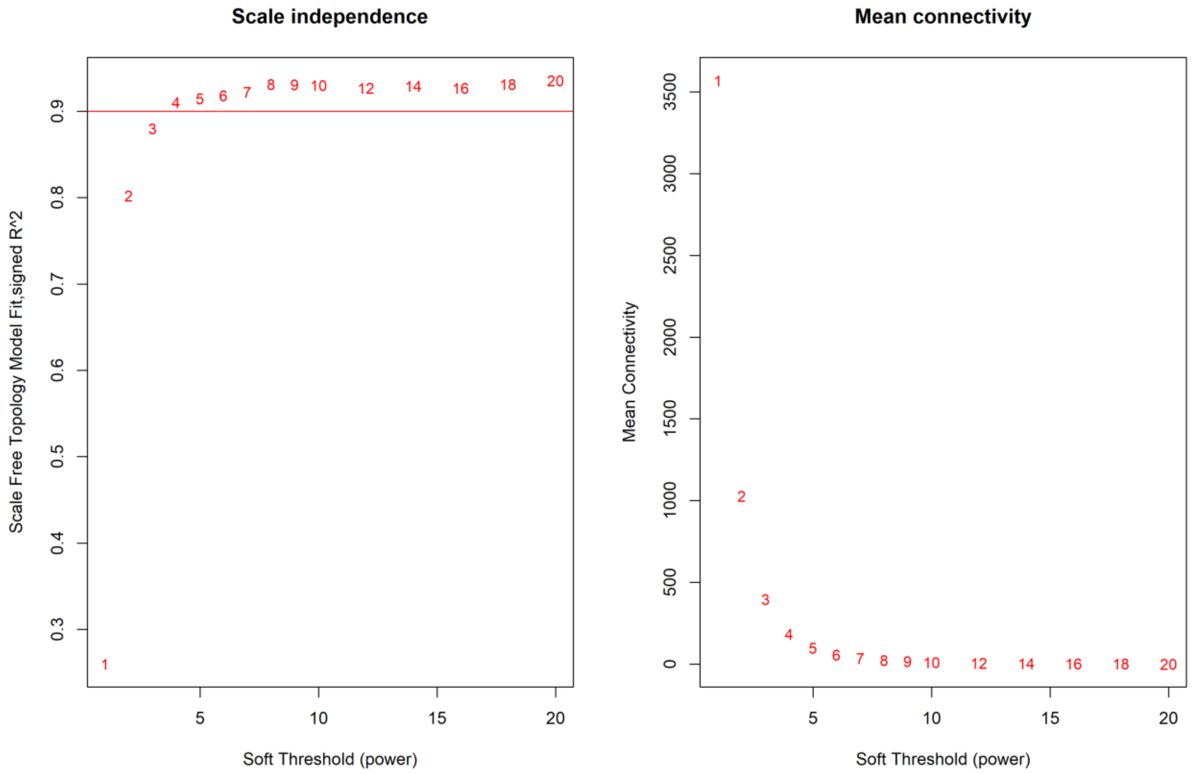

Figure S1. Soft-thresholding powers test to identify the optimized value to fit the approximate scale free topology of the network. The left panel displayed the scale-free topology index (y-axis) with the tested soft-thresholding power (x-axis). The right panel displays the mean connectivity (degree, y-axis) with tested soft-thresholding power (x-axis).

Figure S2

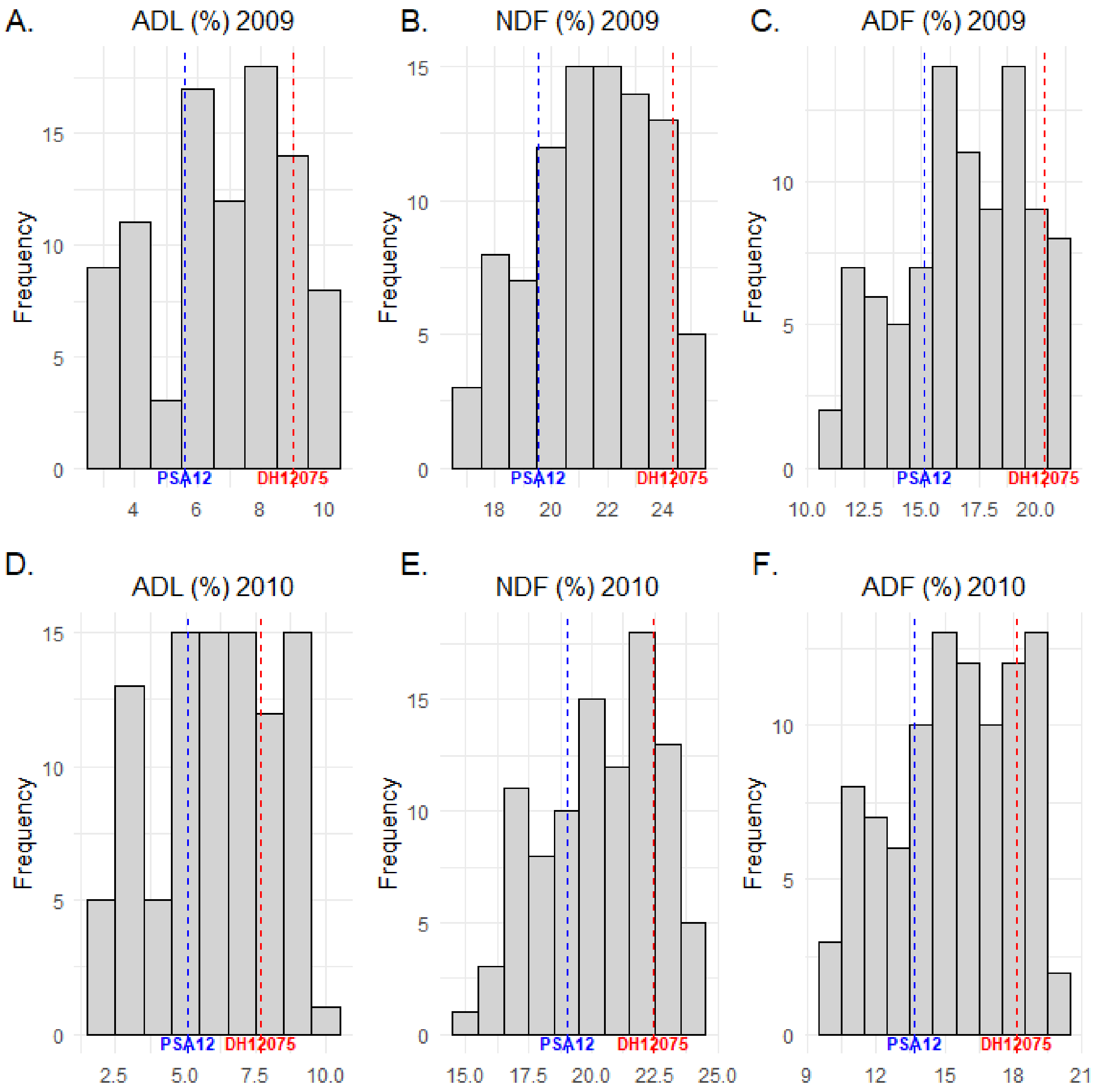

Figure S2. Distribution of lignin content in the years 2009 (A-C) and 2010 (D-F). (A and D) acid detergent lignin (ADL); (B and E) neutral detergent fiber (NDF); (C and F) acid detergent fibre (ADF). Lignin contents of the parental lines PSA12 and DH12075 are highlighted with blue and red dashed lines, respectively. The normality of the distribution was assessed using the Shapiro-Wilk test, which yielded p values of (A) $p = 0.001$, (B) $p = 0.032$, (C) $p = 0.003$, (D) $p = 0.001$, (E) $p = 0.0099$, (F) $p = 0.001$, which all exceed the significance of $p < 0.05$.

Figure S3

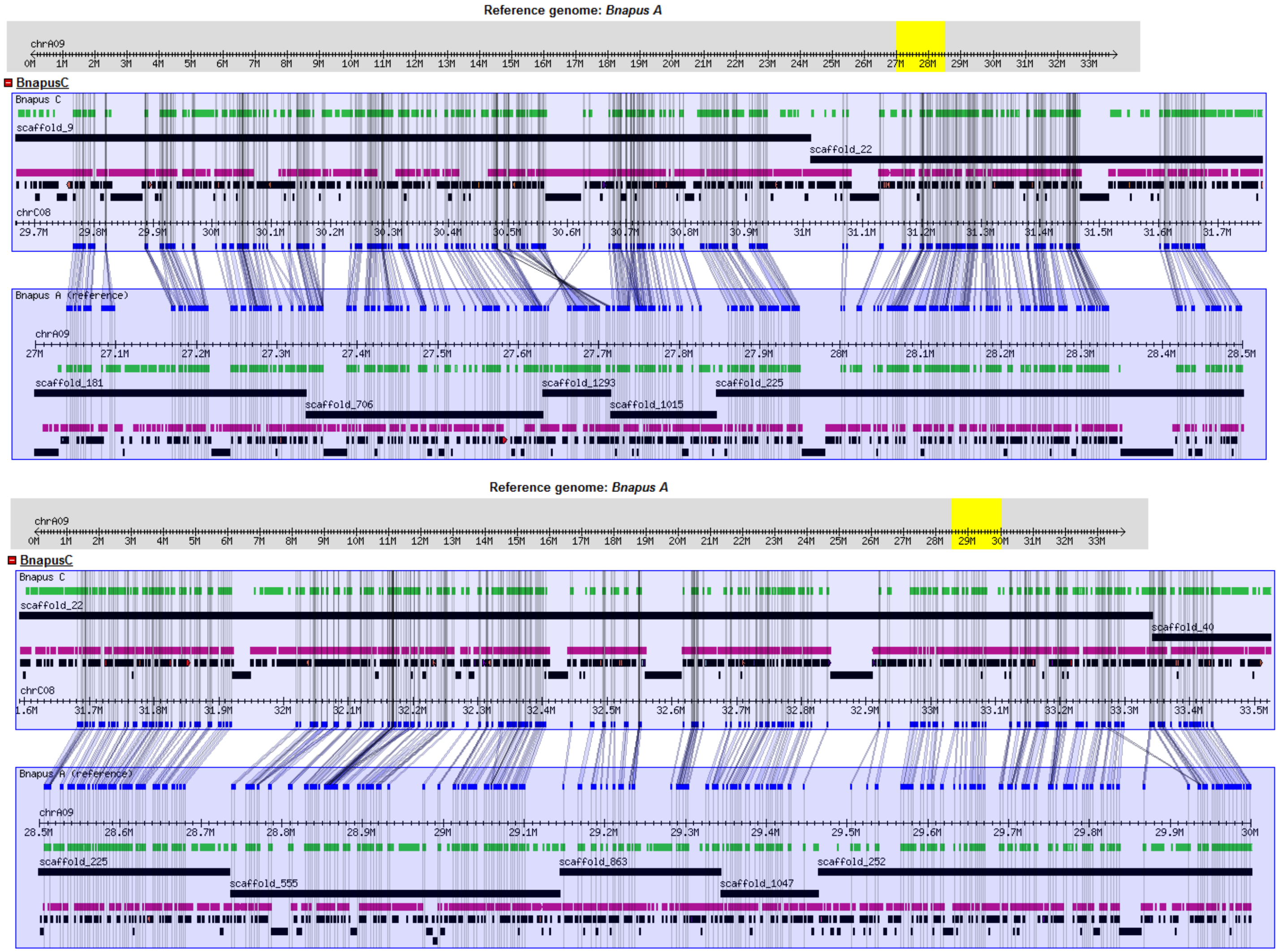

Reference genome: *Bnapus A*
chrA09
BnapusC
Bnapus C
scaffold_9
scaffold_22
chrC08
Bnapus A (reference)
chrA09
scaffold_181
scaffold_706
scaffold_1293
scaffold_1015
scaffold_225
Reference genome: *Bnapus A*
chrA09
BnapusC
Bnapus C
scaffold_22
scaffold_40
chrC08
Bnapus A (reference)
chrA09
scaffold_225
scaffold_555
scaffold_863
scaffold_1047
scaffold_252

Figure S3. Synteny analysis of the lignin QTL qLignin_A09_2 and qLignin_C08. Synteny was analyzed and visualized with the Brassica napus Genome Browser (https://www.genoscope.cns.fr/brassicanapus/) with the Darmor-bzh reference genome (Chalhoub et al., 2014).

Figure S4

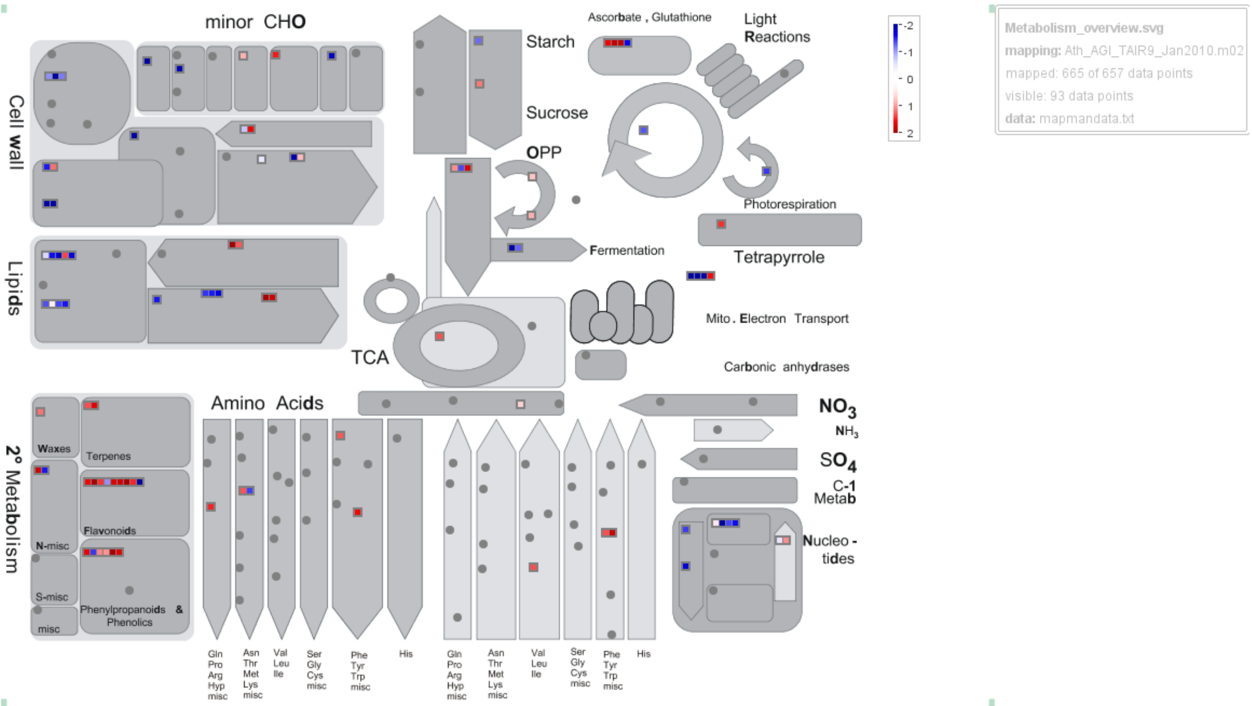

Figure S4. Mapped transcripts from fibre network to the major metabolites map. Correlation between transcripts and seed ADF (acid detergent fibre) trait was presented by colors ranging from red (high positive correlation) to green (high negative correlation).

Figure S5

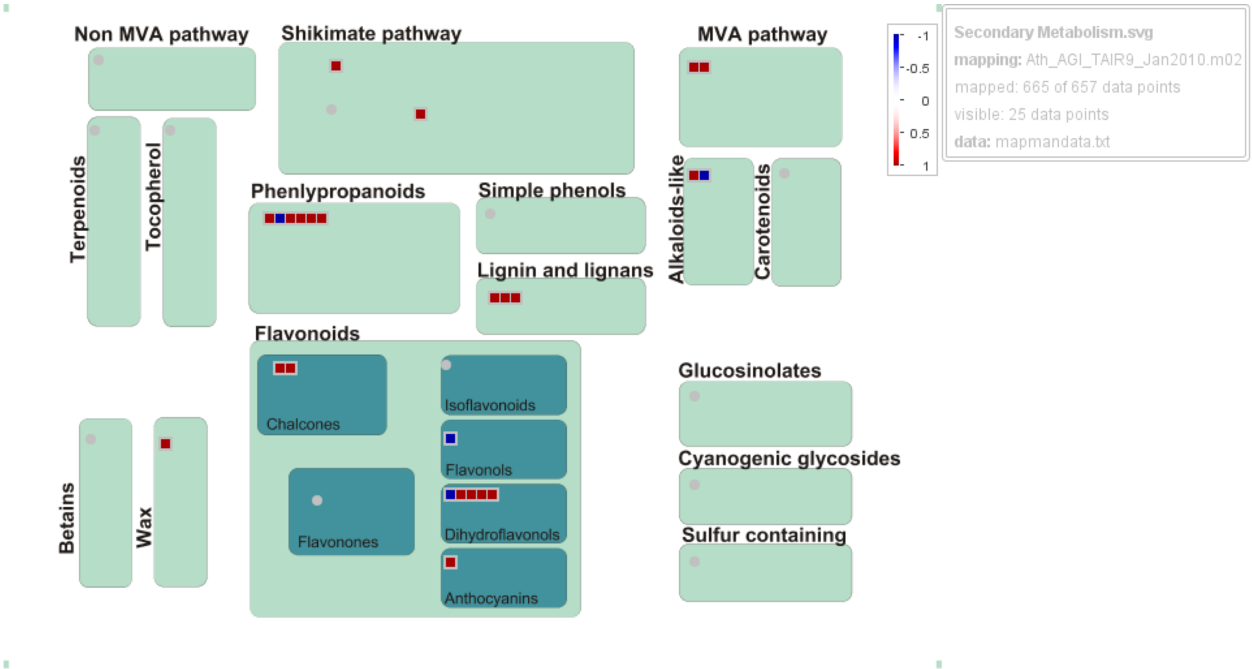

Figure S5. Dissection of transcripts on the major secondary metabolite pathways. Correlation between transcripts and seed ADF (acid detergent fibre) trait was presented by colors ranging from red (high positive correlation) to green (high negative correlation).

Figure S6

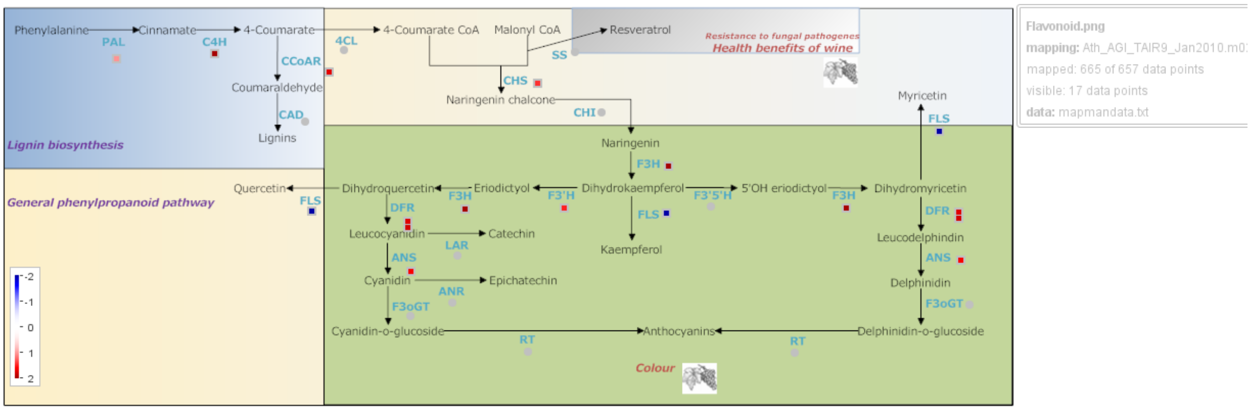

Figure S6. Mapped known genes to the lignin and flavonoid pathway. Correlation between transcripts and seed ADF (acid detergent fibre) were presented by colors ranging from red (high positive correlation) to green (high negative correlation).

Figure S7

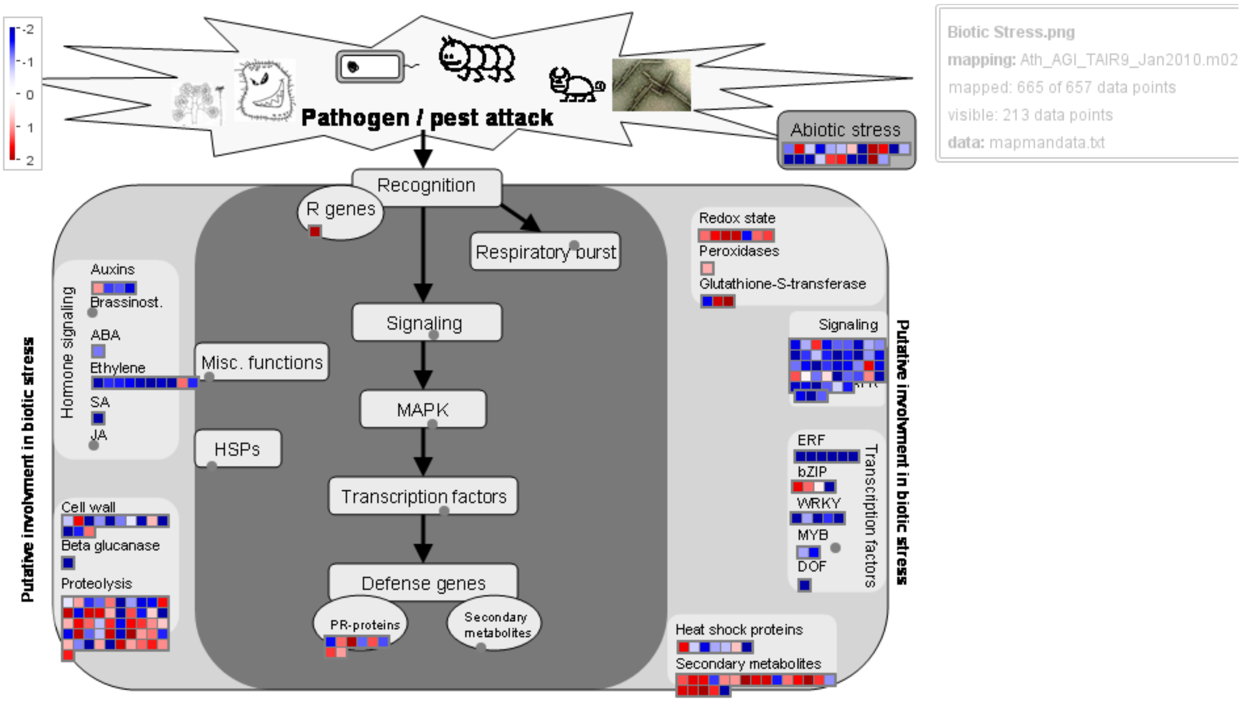

Figure S7. Identification biotic stress signal pathway coordinated with the changes of lignin Correlation between transcripts and seed (acid detergent fibre) trait was presented by colors ranging from red (high positive correlation) to green (high negative correlation).

Figure S8

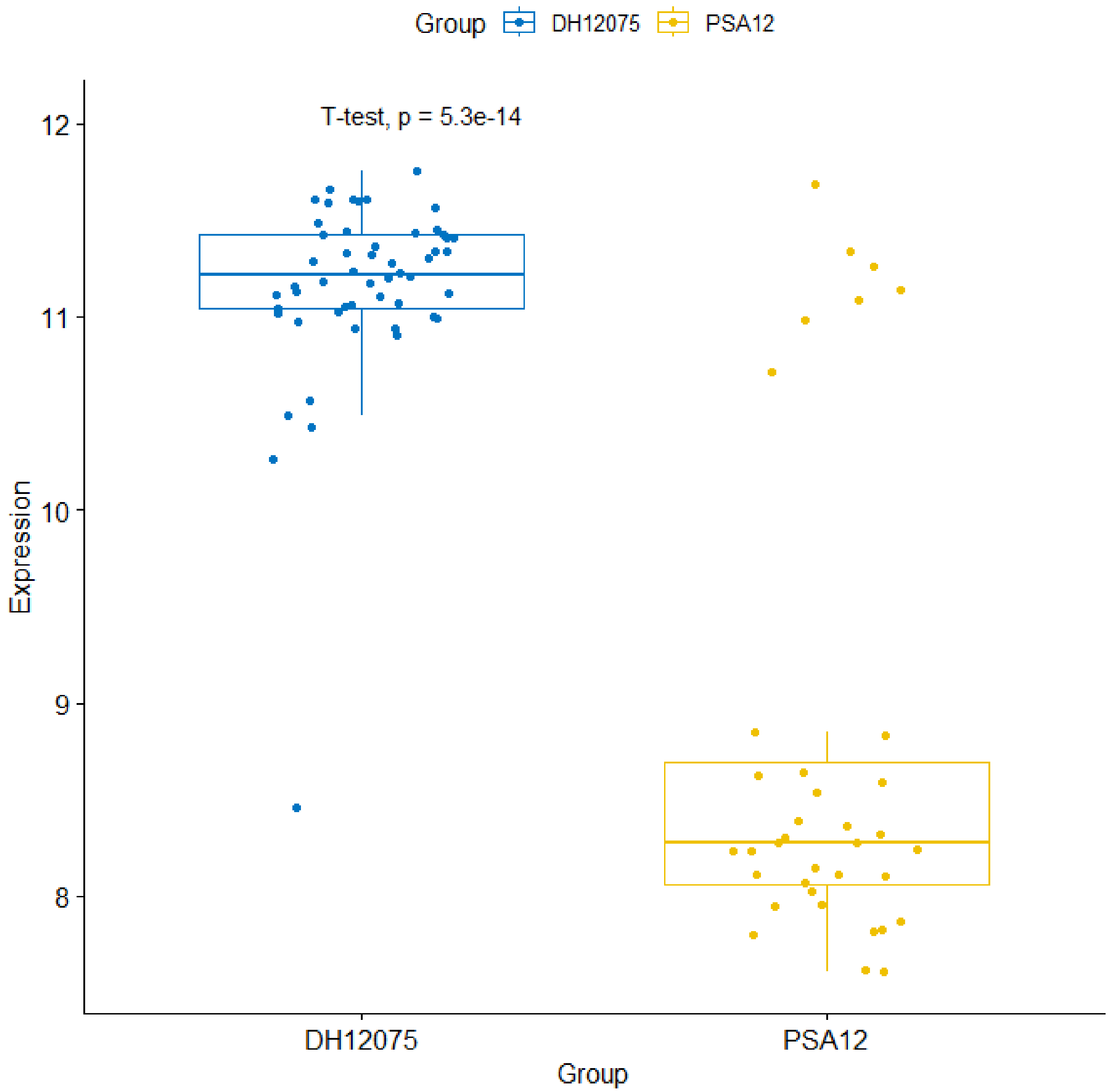

Figure S8. Analysis of gene expression of double haploid (DH) lines with cis-eQTL of *CCR1*. The DH lines were separated into two groups based on the genotype of the eQTL peak marker *BnN9p34992197*: lines with the eQTL (DH12075 marker type) labeled in blue, and lines without the eQTL (PSA12 marker type) labeled with yellow. Student's t-test was used to test the significance of means between groups at the significance level of $p < 0.05$.

Figure S9

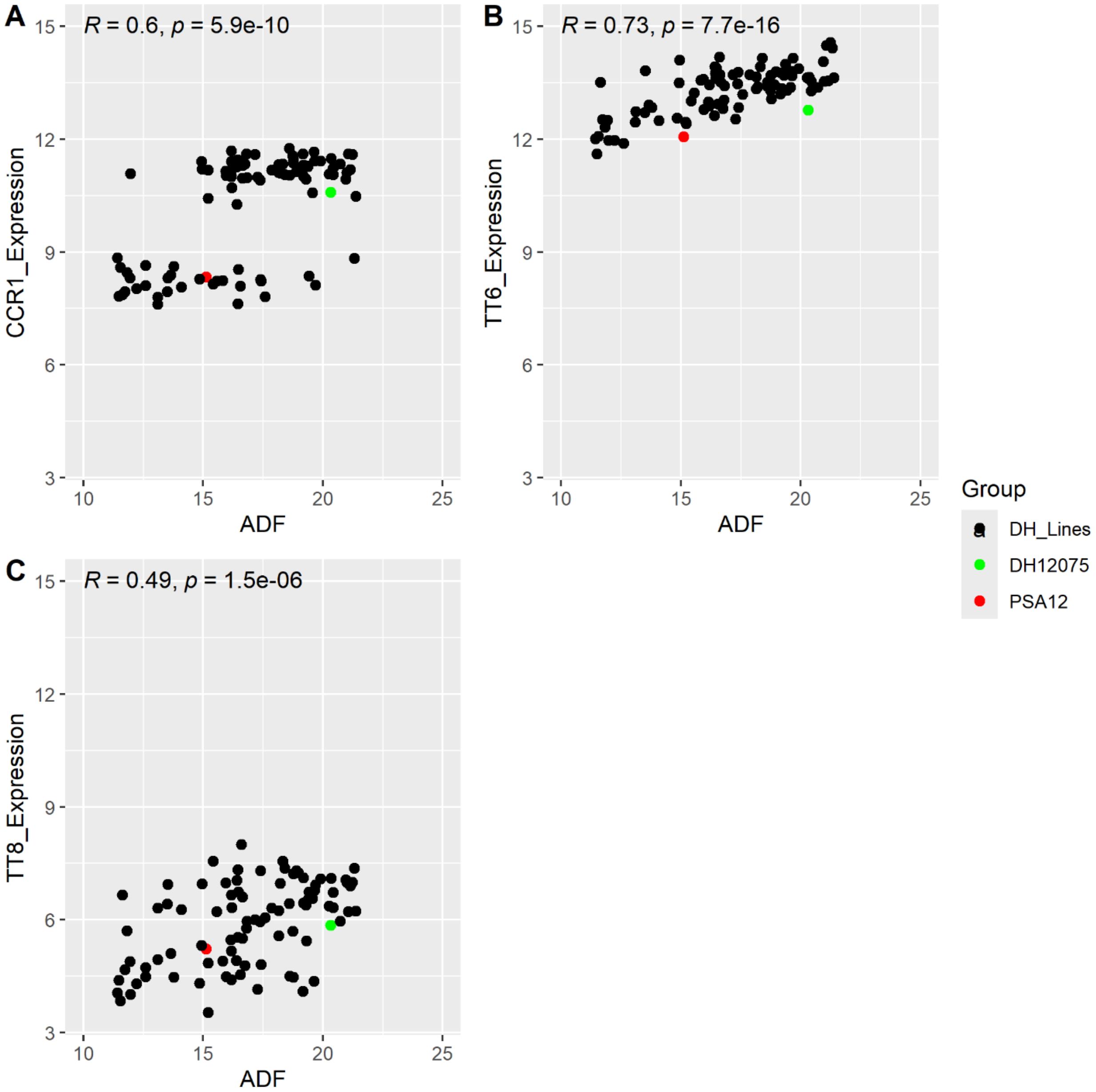

Figure S9. Pearson correlation analysis of trait-gene expression. The log2 transformed gene expression levels of A. *cinnamoyl-CoA-reductase* (*CCR1*); B. *TRANSPARENT TESTA 6* (*TT6*); and C. *TRANSPARENT TESTA 8* (*TT8*) are shown in the Y-axis, with acid detergent fibre (ADF) in the X-axis. Parental line DH12075 is indicated in green, PSA12 is indicated in red, and Double haploid (DH) lines are indicated by black dots. *R,* Pearson Correlation Coefficient; and p, p-value, significance level was claimed at $p < 0.05$.
